# Relative Comparison of Catalytic Characteristics of Human Foamy Virus and HIV-1 Integrases

**Published:** 2009-07

**Authors:** E. S. Knyazhanskaya, M. A. Smolov, O. V. Kondrashina, M. B. Gottikh

**Affiliations:** 1 Chemistry Department of MSU;; 2 Department of Bioengineering and Bioinformatics of MSU;; 3 A. N. Belozersky Institute of Physico-Chemical Biology, M. V. Lomonosov Moscow State University

## Abstract

Due to their ability to integrate into the host cell's genome, retroviruses represent an optimal basis for the creation of gene therapy vectors. The integration reaction is carried out by a viral enzyme integrase: thus, a detailed research of this enzyme is required. In this work, the catalytic properties of human foamy virus integrase were studied. This virus belongs to the Retroviridae family. The dissociation constant was determined, together with the kinetics of integrase catalytic activity. The data obtained were compared to those for the human immunodeficiency virus integrase and a considerable similarity in the activity of the two enzymes was observed.

## INTRODUCTION

Due to their ability to integrate into the genomes of non-dividing cells, retroviruses are widely used as a base for gene therapy vectors construction. A number of papers 1-6 report on systems employing human immunodeficiency virus type 1 integrase (HIV-1 IN) as a basis for the creation of constructs enabling integration of a certain vector into a given DNA sequence. However, directed integration vectors on the basis of HIV carry a potential danger to human health because of their high pathogenicity. In this regard, human foamy virus (HFV), which infects human cells efficiently, but is not pathogenic 7, seems attractive. HFV belongs to the Spumaviridae genus of the retrovirus family and carries an enzyme, integrase (HFV IN), which accomplishes the integration of the viral genome into the host cell's genome. At present, the HFV IN catalytic properties are relatively little-studied. In this paper, an attempt has been made to explore the IN HFV catalytic properties and compare them with those of HIV-1 IN, so as to evaluate the potential for using HFV integrase for sitedirected integration.

**Fig. 1.**
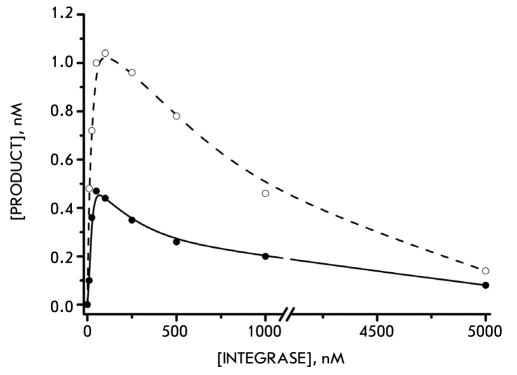
The influence of the HIV-1 (white spots) and HFV (black spots) integrase concentrations on the outcome of the 3'-processing reaction. The integrase solution (5nM - 5µM) was incubated with a 4 nM DNA substrate for 1h at 37 °C

One of the factors hampering the study of the catalytic properties of retroviral integrases is their low activity: to accomplish 3'-processing, a very large excess of the enzyme over DNA is required (usually > 30:1). Therefore, in our study of the HFV IN properties we first explored the dependence of the 3'-processing efficiency on the enzyme concentration in the reaction mixture. To this end, synthetic DNA duplexes imitating the terminal sequence of the U5 domain of the viral DNA's long terminal repeat were employed. Incubation of IN with such DNA-substrate resulted in dinucleotide removal from the 3'-end of the processed strand (U5B-strand). For both Ins, maximum reaction efficiency was achieved at an enzyme concentration of 100 nM Fig. 1(Figure 1). HIV IN's low enzymatic activity is accounted for by the single-turnover mechanism of the catalytic process, the causes for which include the formation of a strong complex between the enzyme and the DNA sequence 8. Therefore, in the next step of our study of the HFV IN properties we explored the DNA-binding stage of the integration process.

**Fig. 2.**
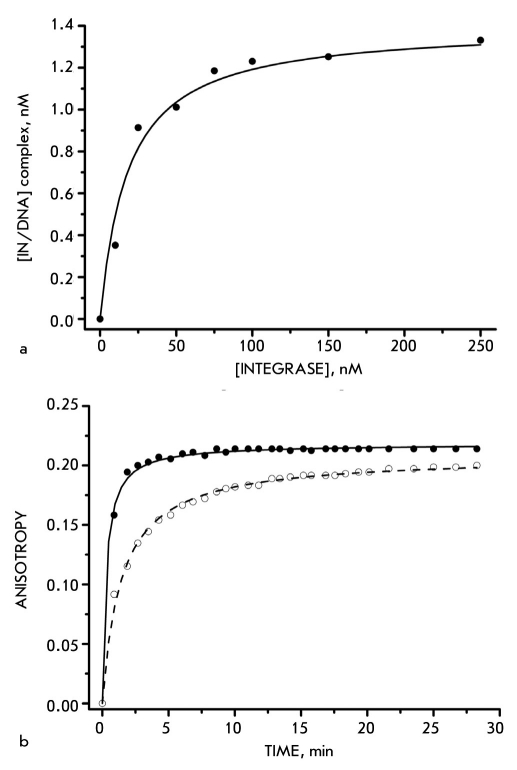
The binding of DNA with HIV and HFV Ins. (a). The isotherm of IN HFV binding a 2nM U5-HFV substrate. The incubation was carried out for 20 min at 25 °C in buffer solution containing 20 mM Tris (pH 7.2), 20 mM NaCl, 1 mM DTT, and a 5 mM MgCl2. The DNA/protein complexes were analyzed by gel retardation assay. (b). The fluorescence polarization assay was applied for constructing the kinetics of the binding of a 4 nM DNA substrate by 100 nM HIV (white spots) and HFV (black spots) integrases at 25 °C

In order to determine the dissociation constant of the HFV IN-DNA complex, we examined 2 or 10 nM DNA substrates binding at different enzyme concentrations (Figure 2A). Application of the approach based on the simple ligandreceptor interaction theory to the system under study allowed employing equation (1)

**Formula 1:** 

to calculate the Kd value, which appeared to be 15-20 nM. This value indicates that the DNA forms a rather stronger complex with HFV IN than with HIV IN (40 nM 8).

We also studied the DNA-binding kinetics of both enzymes by the fluorescence polarization method. The experiment was performed at 25°C, since it is known that under such conditions retroviral INs are capable of associating with their substrates without executing the substrates processing. When the IN solution was added to a fluorescently labeled DNA-duplex, an abrupt increase in the fluorescence signal's anisotropy, conditioned by a slower complex rotation, was observed (Figure 2B). The DNA-HIV IN complex formation is accomplished in 3-4 minutes, which is approximately five times longer than the time required for the DNA association with HFV IN 8. This fact is also indicative of greater favorability of HFV IN binding to the DNA.

**Fig. 3.**
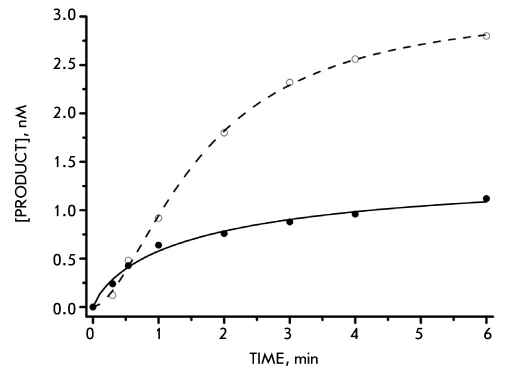
The kinetics of the 3'-processing reaction. The product accumulation curve of a 4 nM U5 substrate 3'-processing reaction while incubated with a 100 nM HIV (white spots) and HFV (black spots) integrases

The results obtained accord well with the data on the time dependence of accumulation of the DNA-substrate's catalytic conversion products. Figure 3 presents the curves corresponding to accumulation of the products of the 3'-processing and strand-transfer reactions catalyzed by the two INs.

It can be seen that the lag-phase, preceding the linear growth phase of product accumulation, characteristic of HIV IN action, is entirely absent in the case of HFV IN. Instead, the process passes straight into the linear growth phase. The calculated stationary rates of product formation at this stage have similar values for both integrases (Vlinear (HIV) = 0.011 nmole/min, Vlinear (HFV) = 0.014 nmole/min) and are remarkably low, which is not typical for a multiple-turnover mode of the enzyme action at the concentrations concerned. It has been shown that the reason for such behavior of HIV INs isolated in the presence of zinc ions and in the absence of detergents according to the procedure in 9 is their low natural catalytic activity rather than low active protein form content in the preparations used. This leads to the inapplicability of the classical Michaelis-Menten formalism to the description of HIV IN's catalytic action.

Instead, one has to employ kinetic equations which assume that the reactions catalyzed by the IN proceed under so-called "single-turnover" conditions, implicating a large excess of enzyme over the substrate 8. The same assumption was made in case of HFV IN. The value of the catalytic constant analogue determined using the "single-turnover" approach appeared to be virtually the same for both enzymes (kcat' = 0.004±0.001 min-1), which also denotes similar properties of both integrases. Moreover, it should be noted that the value of the Michaelis constant analogue computed from kinetic data for HIV IN (Km = 30±5 nM) coincides well with the value of the HIV IN-DNA complex dissociation constant (Kd = 40 nM). At the same time, for HFV IN such correlation is not observed. The calculated Km' = 60±10 nM correlates very poorly with the determined Kd value (15-20 nM). Further we are planning to study the reasons for such a discrepancy.

In conclusion, our results demonstrate that HIV IN is similar to HIV-1 IN in its kinetic characteristics. In the nearest future, we plan to use this enzyme for creating a directed DNA integration system. 

## Experimental section

Oligonucleotides 

U5B≤HIV (5'≤GTGTGGAAAATCTCTAGCAGT-3'),

U5A≤HIV (5'≤ACT GCT AGAGATTCACAC-3'),

U5B-HFV (5'≤ATACAAAATTCATGACAAT-3'),

U5A-HFV (5'≤ATT GTC ATGGAATT GTAT-3')

were synthesized by the amidophosphite method on an automatic DNA synthesizer ABI 394 (Applied Biosystems) according to the standard procedure using commercial reagents (Glen Research) and purified by electrophoresis in 20% polyacrylamide gel containing 7M urea.

Recombinant HIV-1 integrase was expressed in Escherichia coli, isolated and purified without detergent as previously described [9]. HFV integrase was a kind gift of Dr. Mouscadet J-F. (Normal Superior School of Cachan, France).

3'-Processing was performed by incubating the corresponding 4 nM DNA-substrate, containing 5'-[P^32^]-labeled processed strand U5B and HFV IN or HIV IN in 20 ≤L buffer (20 mM Hepes (pH 7.2), 1 mM DTT and 7.5 mM MgCl2), at 37°C. The reaction was arrested by adding 80 ≤L of solution containing 10 mM Tris-HCl (pH 7.5), 0.3 M sodium acetate, 1 mM EDTA, and 0.125 ≤g /ml glycogen; the integrase was extracted with phenol, the reaction products were precipitated with ethanol and resuspended in 80% formamide-water solution. The products were separated by electrophoresis in 20% polyacrylamide gel under denaturating conditions (7M urea) with subsequent gel analysis on a Phosphorimager. The 3'-processing completion was determined by the appearance of a band corresponding to a 2-nucleotide truncated processed strand of the duplex on a radiograph.

Gel-retardation method. [P^32^]-labeled DNA-substrate (2 or 10 nM) was incubated with HFV IN of different concentrations (0-300 nM) in a buffer containing 20 mM HEPES, pH 7.2, 1 mM DTT , 7.5 mM MgCl2, and 5% glycerin at 25°C for 20 min. Afterwards, the mixture was analyzed by electrophoresis in 8% polyacrylamide gel in a buffer containing 20 mM Tris-CH3COOH, pH 7.5, and 7.5 mM MgCl2 at 4-8°C. Gel was analyzed using a STORM 840TM Phosphorimager (Molecular Dynamics). The effective dissociation constant was computed using equation (1).

Fluorescence polarization method. DNA-substrate (4 nM) containing a fluorescein residue in the 5'-processed strand U5B, was incubated with 100 nM HFV IN or HIV IN in 200 ≤L buffer containing 20 mM HEPES, pH 7.2, 1 mM dithiothreitol, and 7.5 mM MgCl2 at 37°C. The fluorescence anisotropy alteration during the incubation was registered using a Cary Eclipse spectrophotometer (Varian).

Computation of the Michaelis constant and the catalytic constant analogues was done in a single-turnover mode using equation (2) according to the previously reported data 8:

**Formula 2:** 

## Acknowledgements

This work was supported by the Russian Foundation for Basic Research (grants 08-04-01252 and 09-04-93107-NCNIL). 
